# In Silico Selection of GAT-1 Inhibitors

**DOI:** 10.3390/ph19071011

**Published:** 2026-06-29

**Authors:** Kristina Stevanovic, Vladimir Perovic, Sanja Glisic, Milan Sencanski

**Affiliations:** 1 Laboratory of Bioinformatics and Computational Chemistry, Institute of Nuclear Sciences Vinca, National Institute of the Republic of Serbia, University of Belgrade, 11001 Belgrade, Serbia; vladaper@vin.bg.ac.rs (V.P.); sanja@vinca.rs (S.G.); 2 Theory Department, National Institute of Chemistry, Hajdrihova 19, 1000 Ljubljana, Slovenia

**Keywords:** GAT-1, SLC6A1, virtual screening, ISM-SM, molecular docking, natural products, EIIP, structural separation, ADMET

## Abstract

The primary control mechanism for synaptic uptake of GABA is through γ-aminobutyric acid transporter 1 (GAT-1, SLC6A1), a known target for anti-epileptic drugs. Although there is a clinically used GAT-1 inhibitor, tiagabine, the development of a new ligand with an advanced pharmacological profile is desirable. For this purpose, a multi-tiered virtual approach to screening has been created, involving pharmacophore-based search; application of the Informational Spectrum Method for Small Molecules, followed by EIIP/AQVN filtering (ISM-SM); molecular docking using an ensemble of several experimentally obtained structures of GAT-1; and ADMET predictions. Pharmacophore-based screening of the ZINC database of natural products, combined with ISM-SM/EIIP filtering, yielded 237 candidate compounds. Structural separation analysis discriminated between the positives and negatives, enabling enrichment-based prioritization. The use of a composite normalized rank score based on docking affinity and structural similarity allowed for the identification of the top candidates: ZINC03643214 and ZINC67840571. Collectively, these refinements establish a more sophisticated computational model for identifying novel GAT-1 inhibitors and highlight promising candidates for future experimental evaluation.

## 1. Introduction

The central nervous system (CNS) has a finely tuned balance between excitatory and inhibitory signaling [1]. The principal inhibitory neurotransmitter in the central nervous system (CNS) of mammals is γ-aminobutyric acid (GABA), which mediates its effects through GABAA, GABAB, and GABAC receptors [2]. Inhibitory signaling through the GABAergic system is mainly regulated at the level of GABA uptake via the four types of GABA transporters (GATs) [1]. The homeostatic equilibrium between excitatory and inhibitory neurotransmission is maintained by regulating precise extracellular GABA levels, and disturbances of this equilibrium are associated with neurological and psychiatric disorders, making GATs important drug targets [3]. Pharmacological inhibition of the GABA transporter GAT-1 increases synaptic and extrasynaptic GABA availability, enhancing phasic and tonic GABAA receptor-mediated inhibition and reducing seizure activity [3,4]. It is reported that approximately 75–80% of synaptic reuptake of GABA is mediated by GAT-1 [5]. In addition, other transporter subtypes cannot functionally compensate for GAT-1’s loss or inhibition; GAT-1 remains the indispensable regulator of inhibitory neurotransmission [6].

GABA is the dominant neurotransmitter at an estimated 60–75% of CNS synapses, which can explain the importance of tight regulation of GAT-1 activity in maintaining overall brain excitability [5].

Also, pathogenic variants in the SLC6A1 gene encoding GAT-1 are associated with a broad spectrum of neurodevelopmental disorders, such as autism spectrum disorder and epilepsy syndromes [6]. Despite the important therapeutic potential of GAT-1, clinical translation remains limited, and to date, tiagabine—a selective GAT-1 inhibitor—is the only approved drug in this class [4,7]. Tiagabine is a derivative of nipecotic acid, with a lipophilic chain attached to the protonated nitrogen of the piperidine ring at one end and two tiophene rings at the opposite end [7]. Historically, nipecotic acid was among the earliest identified GABA reuptake inhibitors and has been used as a prototypical scaffold for the development of selective GAT-1 inhibitors. It is a structural analogue of GABA that possesses key pharmacophoric features necessary for transporter binding, thereby providing a therapeutic rationale for targeting GABA reuptake [8]. Also, nipecotic acid has been shown to exhibit unfavorable pharmacokinetic properties, particularly low permeability across the blood–brain barrier, which limits its therapeutic use. However, its molecular structure has served as a valuable basis for rational drug design, facilitating the development of clinically relevant, high-affinity ligands, such as tiagabine [8]. Numerous attempts to identify novel GAT-1 inhibitors using combined structure- and ligand-based strategies have been documented; however, clinical progress has been hampered by the suboptimal pharmacokinetic profiles of candidates, particularly their inability to cross the blood–brain barrier (BBB), as well as by adverse motor impairments [9,10].

In the search for safer alternatives, natural products have been the basis for medicines in various cultures since ancient times. They are considered safe due to their natural origin and have also been shown to be effective [11]. Medicines of natural origin and their ingredients are considered safer and more effective in terms of toxicity and bioavailability than those derived from non-natural sources [11]. Due to the diverse spectrum of pharmacophores and stereochemistry found in natural product collections, natural products could also be very important in identifying potential new candidates for challenging targets [12]. GAT subtypes possess a high degree of structural homology, complicating selective inhibition, so rigid, stereochemically defined skeletons, which are often features of natural products, would represent a rational strategy to improve GAT subtype discrimination and pharmacological precision [13]. Virtual screening, the in silico equivalent of high-throughput screening of large compound databases, is an essential aspect of the drug development process, significantly reducing the time and cost required to discover new drugs [14]. In this in silico study, we used the ZINC database [15], a freely available 3D molecular library of 20 million commercially available chemicals, to identify novel GAT-1 inhibitors via in silico screening. We screened the ZINC database in silico using a methodology that integrates combined molecular filtering with analyses of both long- and short-range intermolecular interactions. We used established GAT-1 inhibitors as reference templates. Based on our in silico analyses, we propose several molecules as potential candidates for further experimental validation.

Beyond identifying individual candidate molecules, the proposed workflow has broader relevance to rational drug discovery. By integrating pharmacophore-based screening, long-range recognition principles (ISM-SM), structure-based docking, and data-driven prioritisation, the approach provides a systematic framework for reducing large chemical spaces to a small number of experimentally tractable candidates. Such multi-level filtering strategies are particularly valuable in pharmaceutical research, where improving hit quality while minimising experimental cost remains a major challenge. The methodology presented here may therefore serve not only for the discovery of novel GAT-1 inhibitors, but also as a generalizable framework for early-stage ligand identification and rational drug design targeting other membrane transporters and challenging CNS drug targets. Each methodological layer contributes to progressively refining candidate molecules, thereby mimicking the decision-making steps of modern drug discovery pipelines. By focusing on natural products with stereochemically defined scaffolds, our approach not only addresses the pharmacokinetic limitations of earlier GAT-1 inhibitors but also provides a translational framework for identifying ligands with improved safety and efficacy profiles. This structured pathway underscores the relevance of computational strategies as indispensable tools in pharmacy and drug development, bridging theoretical design with experimental validation.

## 2. Results

### 2.1. ZincPharmer Filtering

Nipecotic acid is a valuable scaffold for developing GABA transporter 1 (GAT-1) inhibitors, owing to its structural similarity to gamma-aminobutyric acid (GABA), the primary inhibitory neurotransmitter in the central nervous system. Nipecotic acid closely resembles the structure of GABA, as it contains a nitrogen atom that can mimic GABA’s amine group, which is crucial for binding to the GAT-1 transporter. It has been shown to bind effectively to the GAT-1 transporter. As a result, modifications to nipecotic acid can be designed to increase binding affinity or specificity. Because of these properties, nipecotic acid serves as a starting point for medicinal chemists in designing more effective GAT-1 inhibitors that may have therapeutic applications for conditions like epilepsy, anxiety, and other neurological disorders associated with GABAergic dysfunction [8,9,16].

**Figure 1:**
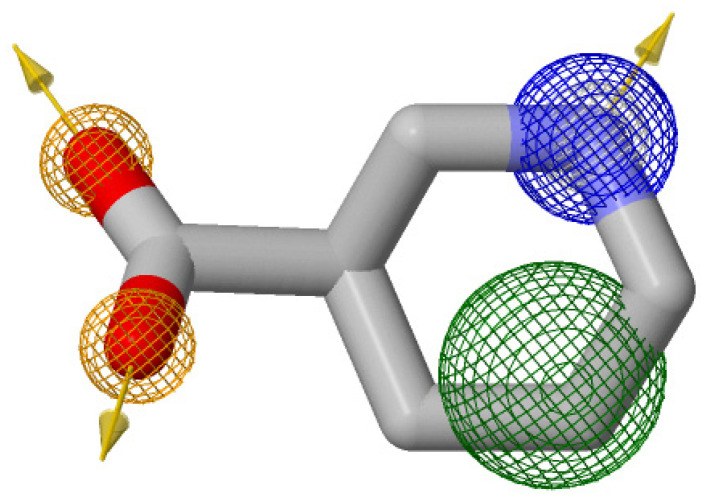
Nipecotic acid as input molecule for virtual screening in ZincPharmer, with marked pharmacophoric classes of the hydrogen bond donors and acceptors (yellow arrows), a positive ion (blue), and a hydrophobic motif (green).

We used the nipecotic acid structure as a feature source (Figure 1). The query was submitted to ZincPharmer [17]. Based on 206,433,075 conformations of 2,177,093 compounds, we obtained 9905 candidates for further filtering.

### 2.2. ISM-SM/EIIP Filtering

In the present study, we have used the Informational Spectrum Method for Small Molecules (ISM-SM) [18,19] for structure/function analysis of the GAT-1 transporter. To determine the most evolutionarily conserved domain, we performed a consensus spectrum (CS) calculation using the human (UniProt code P30531), rat (UniProt code P23978), and mouse (UniProt code P31648) GAT-1 transporters, as all are mammalian. According to Informational Spectrum Method (ISM) principles, the specific IS frequencies that define a biological function are strictly conserved among proteins sharing that function, independent of their sequence similarity [20,21]. This exact methodological approach—using available mammalian orthologs to extract shared, functionally essential informational traits despite limited or varying sequence homology—has been well-established and successfully applied in our previous research [20]. Therefore, the domain determined through these models fully and accurately reflects the core functional characteristics of the human GAT-1 transporter as well.

**Figure 2:**
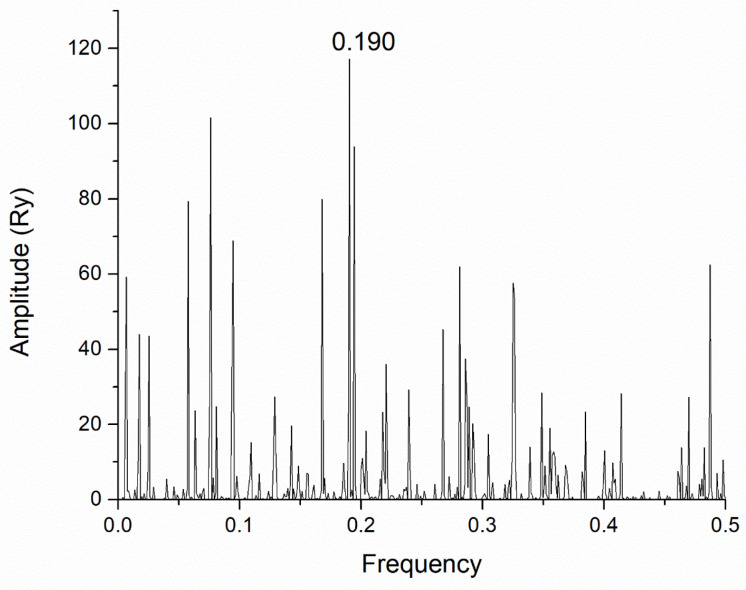
CS of human, rat, and mouse GAT-1 transporters, with the dominant frequency at F(0.190).

**Figure 3:**
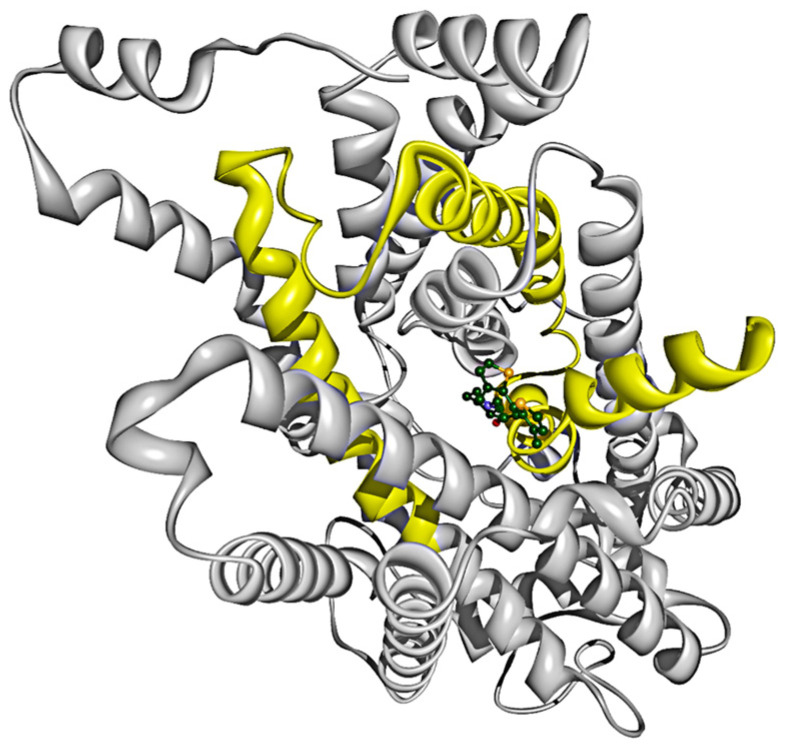
Crystal structure of GAT-1 (PDBID 7Y7Z) with co-crystallized compound tiagabine, with marked recognition domain 14-142 at F(0.190) (yellow).

The consensus spectrum of GAT-1s contains a characteristic frequency at F(0.190) (Figure 2). We select the frequency F(0.190) for further filtering as it is the most selective frequency, given its narrow amino acid region, including residues 14-142, which correspond to the active site domain. This finding is consistent with the co-crystallized inhibitor (Figure 3). With this search, we selected 2155 candidate drugs from the previous step.

We subsequently applied AQVN/EIIP filtering to further narrow the candidate pool. According to the descriptor range of the learning set (2.535714–2.80556, 0.0028515–0.091673) (Supplementary Materials Table S1), applying this to the previous step, we further selected 237 molecules. Molecules falling within this window exhibit electronic properties consistent with those of biologically active ligands from the learning set, thereby enriching the candidate set.

### 2.3. Multi-Conformation Docking and Ensemble Evaluation

To address receptor flexibility and improve the reliability of docking-based prioritization, docking calculations were extended to five experimentally resolved conformations of the human GAT-1 transporter obtained from the PDB database: 7SK2, 7Y7V, 7Y7W, 7Y7Y and 7Y7Z.

Redocking of co-crystallized ligands confirmed the reproducibility of the binding positions for 7SK2–tiagabine (RMSD 0.79 Å) and 7Y7Y–nipecotic acid (RMSD 1.07 Å), while complexes 7Y7W–GABA and 7Y7Z–tiagabine showed higher deviations (>2.6 Å) (Table 1). Docking validation suggests that the 7SK2 and 7Y7Y conformers most reliably reproduce ligand binding, while 7Y7W and 7Y7Z highlight conformational variability that may limit predictive accuracy.

Docking-based discrimination between positive and negative reference compounds was evaluated across five GAT-1 conformations using ROC AUC, the Kolmogorov–Smirnov (KS) statistic, and an enrichment factor of 10% (EF10) (Table 2).

#### 2.3.1. Performance of Individual Receptor Conformations

Docking performance varied substantially across receptor conformations (Table 2). Among the five evaluated structures, 7SK2 and 7Y7Z achieved the highest discrimination between active and negative compounds, with ROC AUC values of 0.726 and 0.727, respectively, and strong KS statistics (0.525 and 0.490).

In contrast, conformation 7V7W showed near-random performance (AUC = 0.457), indicating limited suitability for virtual screening in this context. While some conformations (e.g., 7Y7V) exhibited higher early enrichment (EF10), this was not accompanied by strong global discrimination, suggesting localized ranking effects rather than consistent separation.

This performance-based ranking was further supported by structural considerations, as 7SK2 and 7Y7Z represent receptor states that are more compatible with ligand binding modes relevant to the active reference compounds. Namely, these structures that contain tiagabine are a better fit for candidate screening due to the amino acid residue conformation in the binding site, providing larger binding site volume and less steric hindrance due to the ligand’s size. Taken together, both statistical and structural evidence supported the selection of these two conformations for downstream ensemble docking.

#### 2.3.2. Two-Conformation Ensemble Improves Discrimination

**Figure 4:**
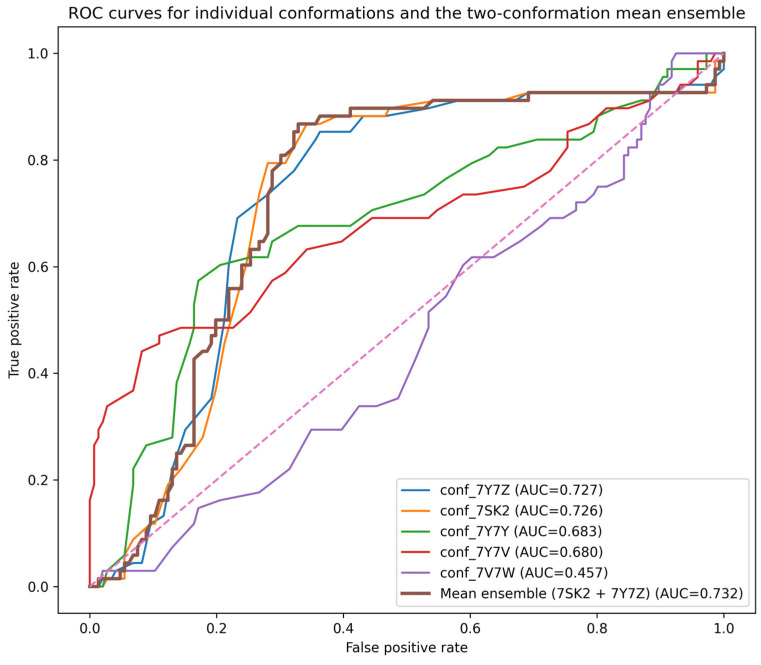
ROC curves for individual receptor conformations and the two-conformation ensemble. Receiver operating characteristic (ROC) curves for five GAT-1 conformations and the mean ensemble of the two selected conformations (7SK2 and 7Y7Z). The ensemble demonstrates improved or comparable discrimination relative to individual conformations.

The mean ensemble of the two best-performing conformations (7SK2 and 7Y7Z) achieved improved performance compared to individual structures, with ROC AUC = 0.732 and KS = 0.539 (Table 2, Figure 4).

This result demonstrates that incorporating limited conformational diversity can enhance predictive performance without introducing additional complexity. The simple mean aggregation proved sufficient to capture complementary information between conformations.

Although the ensemble ROC curve does not dominate all individual curves at every threshold, it exhibits the highest overall performance and more robust behavior across the full operating range (Figure 4). This indicates that the ensemble approach effectively stabilizes docking predictions and reduces sensitivity to individual conformational biases.

Importantly, the purpose of the ROC analysis was not to establish a standalone predictive model of GAT-1 activity, but rather to identify receptor conformations capable of enriching active compounds and supporting candidate prioritization. The observed AUC values therefore served primarily as a criterion for receptor conformation selection and protocol validation. Subsequent candidate ranking integrated docking-derived information with graph-based structural enrichment metrics, reducing dependence on the predictive performance of any single docking model.

**Figure 5:**
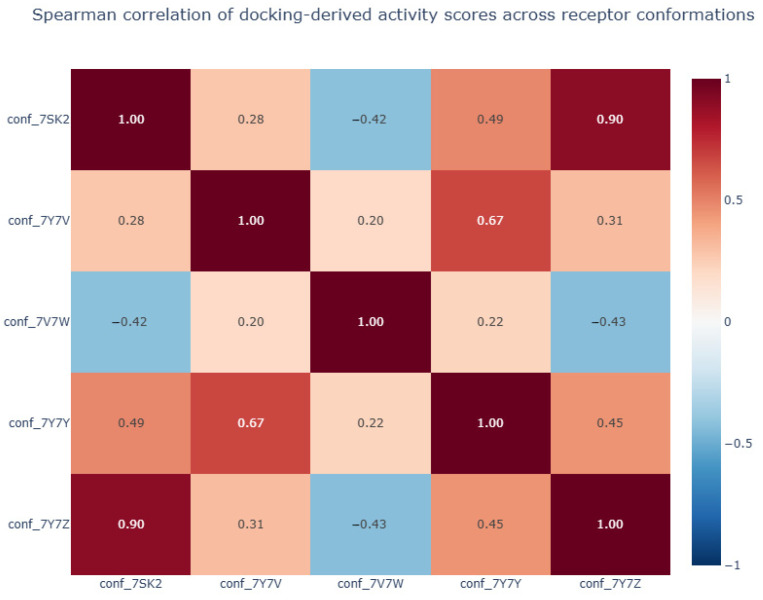
Spearman correlation of docking scores across receptor conformations. Heatmap of pairwise Spearman correlation coefficients between docking-derived activity scores across five receptor conformations. The high correlation between 7SK2 and 7Y7Z supports their joint use in the ensemble.

Pairwise Spearman correlations between docking scores revealed moderate relationships between most conformations (ρ ≈ 0.20–0.67), with the highly correlated pair (ρ ≈ 0.90) being 7SK2 and 7Y7Z, while others showed weak or even negative correlations (ρ ≈ −0.4) (Figure 5). This confirms that receptor conformations capture partially distinct aspects of ligand binding, supporting the rationale for consensus scoring.

### 2.4. Structural Separation Between Positive and Negative Sets

Structural separation between positive and negative molecules was evaluated using pairwise distance analysis. Inter-class and intra-class distances were computed and summarized using separation metrics, as described in Section 4. This is an important part of the filtering process, due to the need for high selectivity towards GAT-1 over other members of the SLC6A family (dopamine, serotonin, and noradrenaline transporters).

Table 3 reports silhouette coefficients, inter–intra distance ratios, normalized inter–intra differences, and Kolmogorov–Smirnov statistics for fingerprint-based and descriptor-based representations.

Cosine distance provided the strongest global separation, while Jaccard distances showed moderate separation, and Euclidean distance showed weaker global separation. These values reflect structural overlap between active and inactive compounds. Cosine distance exhibited the strongest global separation (silhouette = 0.123), followed by Jaccard distances (0.064–0.071), while Euclidean distance showed weaker global separation (0.029). These results indicate partial overlap between positive and negative compounds in the chemical descriptor space, consistent with the heterogeneous nature of chemical activity landscapes. Although absolute separation values are moderate (silhouette = 0.123 for cosine distance), they consistently indicate that positive and negative compounds occupy partially distinct regions of descriptor space. Such moderate separation is expected in chemically heterogeneous datasets and reflects realistic activity landscapes rather than artificially clean class boundaries.

Higher inter-class distances relative to intra-class distances indicate clear structural distinction between positive and negative molecules. The inter–intra ratio and normalized separation confirm that positive and negative sets occupy distinct regions of structural space. The silhouette coefficient indicates modest but consistent separation between positive and negative compounds, reflecting partial structural overlap in the descriptor space. The Kolmogorov–Smirnov statistic further confirms a measurable difference between inter-class and intra-class distance distributions, confirming consistent structural differentiation between positive and negative sets.

Based on the separation analysis summarized in Table 3, cosine distance computed on continuous molecular descriptors was selected as the structural similarity metric for subsequent candidate enrichment and composite ranking. Among all evaluated metrics, cosine distance yielded the highest silhouette coefficient (0.123) and the largest Kolmogorov–Smirnov statistic (0.173), while also demonstrating strong inter–intra separation ratios. This indicates that cosine distance provides the most consistent global discrimination between positive and negative reference compounds in descriptor space; it was therefore chosen as the primary structural enrichment metric.

### 2.5. Candidate Prioritization by Docking Ensemble Score Analysis and Structural Enrichment

Candidate molecules were prioritized by integrating docking ensemble scores with structural enrichment (gain_mean). Docking scores were derived from the two-conformation mean ensemble (7SK2 and 7Y7Z), while gain_mean was computed using cosine-based similarity, which showed the strongest separation between positive and negative reference sets. While docking captures binding affinity, structural enrichment reflects similarity to known active compounds. The combination of these two signals enables more robust prioritization by integrating physicochemical interaction potential with structural knowledge.

A normalized rank-based composite score was used to combine these two components, assigning equal weight (w = 0.5) to docking and structural enrichment. This approach enables balanced prioritization by considering both predicted binding affinity and similarity to known active compounds.

Docking energies across candidates ranged from −12.1 to 16.1 kcal/mol, reflecting varying binding affinities within the ensemble docking framework. Structural enrichment scores (gain_mean), based on cosine similarity to the positive reference set, ranged from −0.204 to 0.181, with higher values indicating greater similarity to known active compounds.

Given that most molecules in the dataset had a limited number of conformers (93.7% ≤ 5, median = 2), top-five aggregation provides a representative summary of conformational variability for the majority of candidates. Due to its balance between robustness and conformational representativeness, top-five conformer aggregation was selected as the primary docking representation for final candidate prioritization.

**Figure 6:**
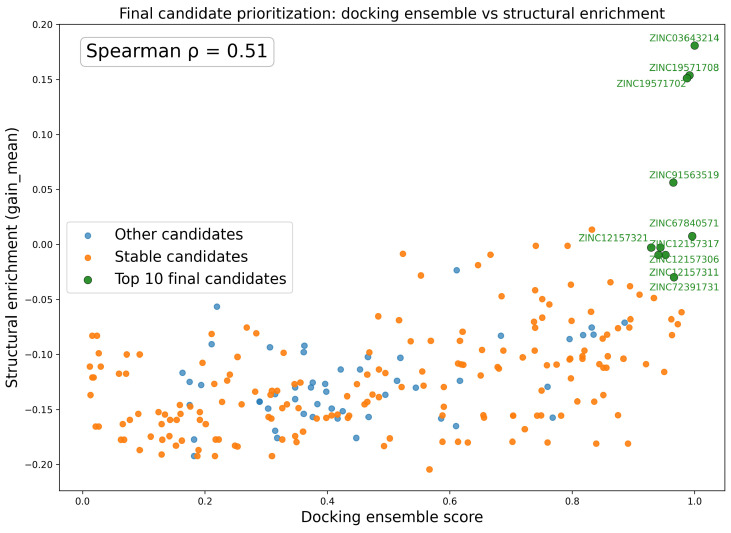
Joint distribution of docking ensemble score and structural enrichment. Scatter plot of conformational docking ensemble score versus structural enrichment (gain_mean) for all screened candidates. Candidates classified as stable across conformer aggregation strategies (rank_range ≤ 10) are shown in orange, whereas the highest-ranked final candidates are additionally highlighted in green and labeled individually. The Spearman correlation coefficient between docking ensemble score and structural enrichment is indicated within the plot.

After combining conformationally ensembled docking scores with structural enrichment toward the positive reference set, a final consensus ranking of candidate molecules was obtained (Table 4, Supplementary Table S2). This integration allowed candidates to be prioritized jointly by predicted binding compatibility across receptor conformations and by structural proximity to known active compounds. Joint distribution of docking ensemble score and structural enrichment of all candidates is presented in Figure 6 and Supplementary Table S3.

A compact set of top candidates emerged from this analysis. The highest-ranked molecules were ZINC03643214, ZINC19571708, ZINC19571702, ZINC67840571, ZINC91563519, ZINC12157317, ZINC12157306, ZINC72391731, ZINC12157321, and ZINC12157311. All top 10 candidates were classified as stable across conformer aggregation strategies, confirming the robustness of the final prioritization. The most robust candidates were ZINC03643214, which was ranked first across all aggregation strategies, followed by ZINC19571708, ZINC19571702, ZINC67840571, and ZINC91563519, all of which showed minimal rank variation. These compounds therefore represent the most reliable consensus hits identified in the present study.

**Figure 7:**
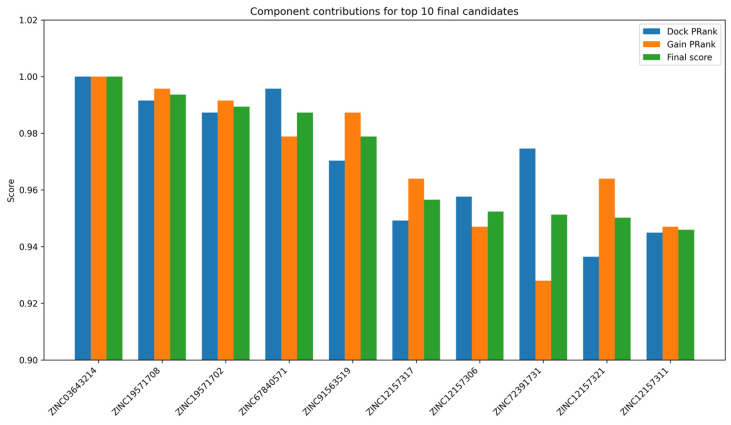
Component contributions to the final consensus ranking. Normalized-ranked docking ensemble score, normalized-ranked structural enrichment score, and final composite score for the top-ranked candidate molecules. The figure illustrates that the top candidates are jointly supported by both docking and similarity-based prioritization, rather than by a single dominant component.

To further illustrate the contribution of each component to the final prioritization, we present in Figure 7 the normalized docking, enrichment, and final composite scores for the top-ranked candidates.

The highest-ranked candidates consistently showed strong performance in both components, rather than being driven by a single extreme score. In particular, the top candidates occupied the region of high docking ensemble score and positive structural enrichment, indicating balanced optimization of both criteria. This supports the use of a composite percentile-rank framework for candidate prioritization, as docking and structural enrichment capture related but non-identical aspects of ligand plausibility.

### 2.6. Robustness of Candidate Ranking Across Conformer Aggregation Strategies

**Figure 8:**
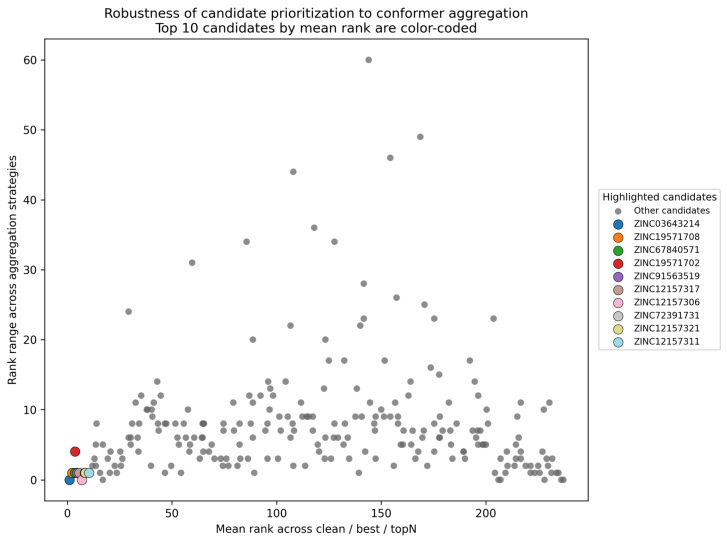
Robustness of candidate prioritization to conformer aggregation. Scatter plot of mean rank versus rank range across the clean, best-conformer, and top-five aggregation strategies. Candidates located in the lower left region combine strong final prioritization with low rank variability and therefore represent the most robust consensus hits.

To evaluate the impact of candidates’ conformer aggregation on candidate prioritization, three docking representations were compared: (i) cleaned single-score representation, (ii) best-conformer aggregation, and (iii) top-five conformer averaging. The results are presented in Table 5 and Table 6, and Figure 8.

The resulting candidate rankings were highly consistent across all three approaches. Pairwise Spearman rank correlations were extremely high (ρ = 0.988–0.996), indicating that the relative ordering of candidates is largely invariant to the choice of conformer aggregation strategy (Table 5). Similarly, strong Pearson correlations (r ≈ 0.991–0.997) were observed for the final composite scores (Table 5).

Analysis of top-ranked candidates further confirmed this robustness. The overlap between top-ranked subsets was substantial, with Jaccard indices ranging from 0.67 to 1.00 across different top-K thresholds (Table 6). In particular, the top-ranked candidates were largely shared across all aggregation methods.

Importantly, a core subset of top-ranked candidates exhibited particularly stable behavior, characterized by low rank variability across aggregation methods. These stable candidates represent the most reliable predictions, as their prioritization is supported by multiple independent representations of conformational space, as shown in Figure 8. In contrast, a smaller subset of candidates exhibited higher sensitivity to the aggregation strategy, showing substantial rank fluctuations.

Overall, candidate rankings showed high consistency across these strategies, indicating that prioritization was not strongly dependent on the specific handling of conformers.

Complete ranking results, including all candidates and detailed ranking statistics across aggregation strategies, are provided in Supplementary Tables S2–S5.

### 2.7. Stability of Top-Ranked Candidates

**Figure 9:**
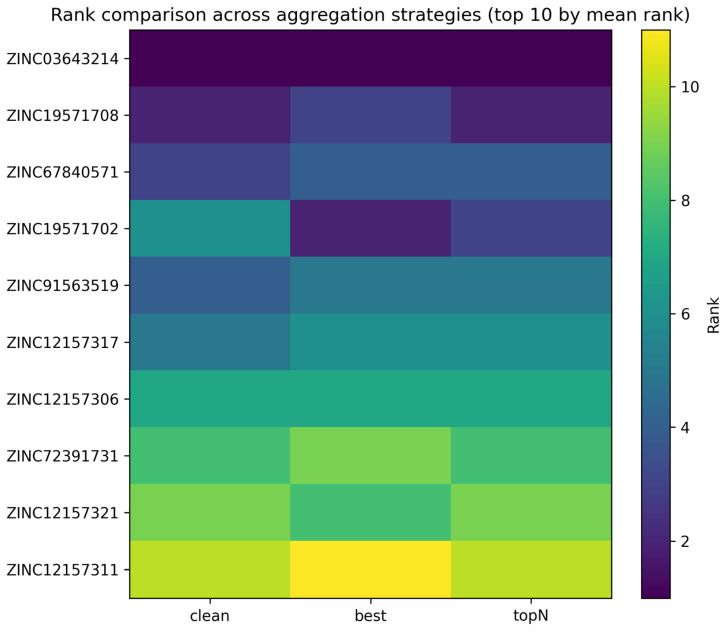
Rank comparison across conformer aggregation strategies for top candidates. Heatmap of final candidate ranks obtained using the cleaned representation, best-conformer aggregation, and top-N conformer averaging. Similar rank patterns across methods indicate stability of the top-priority candidates.

To distinguish robust candidates from aggregation-sensitive ones, the final rankings obtained from the clean, best-conformer, and top-five aggregation schemes were compared. A core subset of candidates remained consistently highly ranked across all three strategies, with minimal variation in final rank (Figure 9, Supplementary Table S4). These molecules can therefore be regarded as stable consensus candidates.

The most stable candidates combined three desirable properties: (i) strong ensemble docking performance across receptor conformations, (ii) favorable structural enrichment toward the positive reference set, and (iii) low sensitivity of their final rank to the specific conformer aggregation method. Taken together, these criteria identify the most reliable molecules for downstream experimental evaluation.

### 2.8. Final Candidate Selection

**Figure 10:**
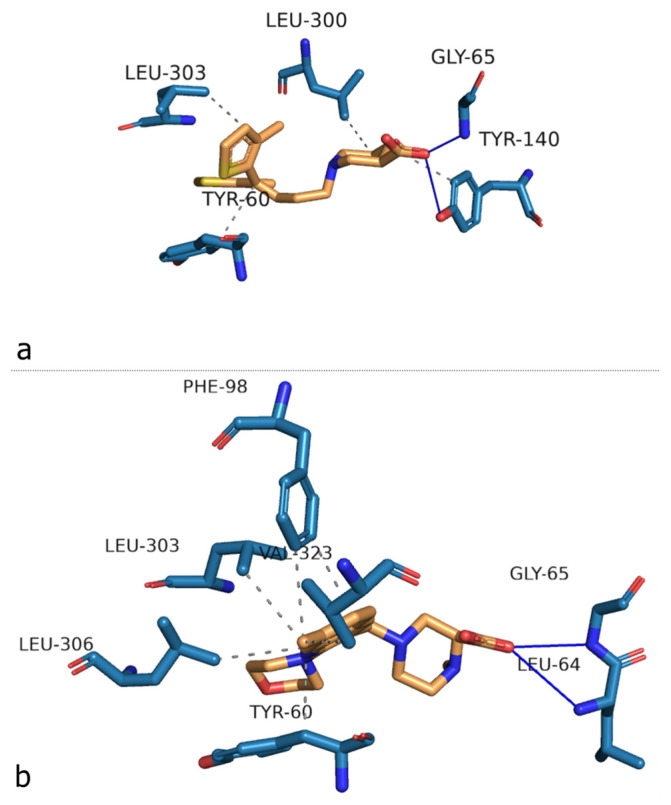
The co-crystallized inhibitor tiagabine in the binding site of GAT-1 (**a**) and the best docked conformation of ZINC67840571 (**b**) in PDBID 7SK2, according to Protein–Ligand Interaction Profiler. Blue lines: hydrogen bonds; gray dashed lines: hydrophobic interactions.

The final ranking identified a set of top candidates combining strong docking support and favorable structural enrichment (Table 4, Supplementary Table S2). To properly analyze and classify potential GAT-1 inhibitors, the binding pattern of tiagabine had to be adopted as a reference. Tiagabine occupies an orientation in the binding site of GAT-1 as follows: the zwitterion moiety is oriented towards the inner receptor space, where the carboxylic residue forms a hydrogen bond with GLY65, while the protonated nitrogen atom is pointed towards SER396. On the contrary, the hydrophobic moiety, consisting of two thiophene rings, is oriented towards the outer receptor space, forming interactions with CYS399, LEU306, and TYR60 (Figure 10a). Notably, the highest-ranked compound (ZINC03643214) corresponds to a previously reported GAT-1 inhibitor candidate [22], providing external validation of the proposed approach. The next-ranked compounds ZINC19571708 and ZINC19571702, while fulfilling the criteria for the second best-ranked compounds and putative best candidates, could not have been considered. Namely, after inspection of the docking positions across all structures, the candidate compounds could not match the tiagabine binding pattern in its docked orientation at the GAT-1 binding site. Therefore, the compounds were considered false positives, and the next-ranked compound (ZINC67840571) was selected as a top-ranked novel candidate. Its high composite score, combined with ranking stability across aggregation strategies, supports its prioritization for further experimental validation.

**Figure 11:**
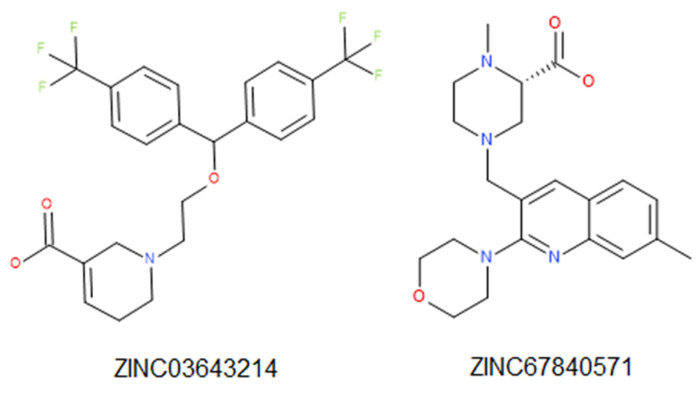
Structures of the compounds from Table 7.

The intermolecular interactions between the GAT-1 binding site and the candidates (Figure 11) are qualified using Protein–Ligand Interaction Profiler [23] (Table 7).

The best hit in our results was the previously discovered GAT-1 inhibitor ZINC03643214, with a reported Ki of 440 nM [22].

### 2.9. ADMET Calculations

To further assess the drug-likeness of the selected compounds, pharmacokinetic calculations were performed for two sets of compounds: ChEMBL database compounds and ZINC database-identified candidates (Supplementary Table S1). ADMET properties were calculated using pkCSM (https://biosig.lab.uq.edu.au/pkcsm/prediction, accessed on 1 March 2026), with 36 variables.

The resulting data were subjected to k-means clustering. The number of clusters was initially set to between 2 and 5, with the clustering criterion based on the determinant (W). Initial partitions were randomized with 10 repetitions, and clustering was stopped after 500 iterations or upon convergence at 0.00001. To determine the optimal number of clusters, an elbow analysis was performed by plotting the within-cluster sum of squares against the number of clusters. The within-class variance was 28.05%, with a between-class variance of 71.95%. Based on the class centroids, the three clusters can be interpreted as follows, according to their distinguishing features:

Cluster 1 consists of molecules with mean molar weight ≈400 and the highest lipophilicity (logP ≈ 3.8), BBB permeability (≈0.07), and CNS permeability (≈−2.04) among all three clusters. These compounds also exhibited favorable Caco-2 permeability (1.086), high intestinal absorption (mean 90%), and high total clearance (logCLtot = 0.433 mL/min/kg). No significant toxicity is predicted, except for hERG I (compounds ZINC19571702 and ZINC19571708) and II (ZINC26963329). In other words, only 0.84% and 0.42% of the candidate compounds report predicted affinity towards hERG I and hERG II, respectively. The cluster contains most of the ChEMBL-reported GAT-1 inhibitors. Twenty ZINC candidates belong to this cluster. Regarding the best candidates, both ZINC03643214 and ZINC67840571 are in this cluster. The central compound of the cluster is CHEMBL3398501. Members of this group represent the most conventional “drug-like” space.

Both clusters 2 and 3 comprise lighter molecules, with molecular weight ≈ 230–285, significantly lower lipophilicity (logP ≈ −0.85, −0.28), and lower BBB permeability (≈−0.194, −0.180). CNS permeability is also lower (≈−3). These compounds exhibit fair intestinal absorption (≈70%) and are negative for Caco2 permeability. Regarding toxicity predictions, neither cluster reports alarming values. Total clearance in both clusters is higher (logCLtot ≈ 0.78 mL/min/kg) than in cluster 1 (0.433 mL/min/kg). All data on ADMET calculations and k-means clustering are available in the Supplementary Materials.

## 3. Discussion

GAT-1 (SLC6A1) is the principal regulator of synaptic GABA reuptake and a validated therapeutic target in epilepsy and neurodevelopmental disorders. Despite its central physiological role, tiagabine remains the only clinically approved selective inhibitor, underscoring the need for new ligands with improved pharmacokinetic and safety profiles. In this study, we implemented a multi-layered in silico workflow integrating pharmacophore screening, ISM-SM/EIIP filtering, molecular docking, structural enrichment analysis, and ADMET profiling to identify novel candidate inhibitors.

A defining feature of this strategy is the combination of long-range interaction compatibility filtering with structure-based docking. The Informational Spectrum Method identified a conserved dominant frequency (F(0.190)) in GAT-1, consistent with preservation of a functional interaction domain. Cross-spectrum filtering of small molecules against this frequency reduced the candidate pool prior to docking.

Docking into the inward-open cryo-EM structures of human GAT-1 identified several candidates with predicted binding energies stronger than that of tiagabine. The top-ranked compounds reproduced key interactions observed for tiagabine, including hydrogen bonding with GLY65, as well as hydrophobic contacts involving TYR60 and LEU303. Other shared interactions between ZINC03643214 and ZINC67840571 include hydrophobic interactions with PHE98 and LEU306. Inspection of the docking outputs discarded false positives that could act as good candidates, however.

ZINC3643214 belongs to the class of diaryl-substituted nipecotic acid derivatives previously described as GAT-1 inhibitors [16,22]. We further searched the natural origin of the proposed compounds in several specialized repositories, including NPBS (Natural Products and Biological Resources) [24] and the KNApSAcK family database [25]. We did not find any NPBS data for any of the selected compounds. We further explored the KNApSAcK family database [25], which provides information on compounds, including family details and plant species of origin. Still, we did not find a matching entry for the proposed candidates. Still, the historical and contemporary role of natural products in GAT-1 research is documented. Guvacine, a naturally occurring alkaloid, provided early evidence that structurally constrained plant-derived compounds can target the GABAergic system [9,26]. Subsequent pharmacological profiling identified it as a selective GABA uptake inhibitor—demonstrating high specificity for the GAT family (GAT-1, GAT-3, and GAT-4) while exhibiting minimal direct activity at GABA receptors [27]. Also, other naturally derived compounds have been published, and their derivatives have already been identified as potential GAT-1 modulators. The lignan (−)-quinoquinine, isolated from *Piper* species, has been shown to inhibit GAT-1 and effectively modulate its kinetic parameters [28]. In addition to the plant world, the structural diversity of natural toxins has also provided promising leads; low-molecular-weight acylpolyamines from the venom of the spider *Parawixia bistriata* have been shown to inhibit GABA uptake in synaptosomes, further supporting the potential of natural small molecules as GAT-1 modulators [29].

Structural separation analysis between positive and negative reference compounds revealed measurable differentiation in descriptor space. Cosine distance provided the strongest discrimination, indicating that angular similarity in continuous descriptor space is most informative for this dataset. The moderate silhouette values are consistent with the known chemical heterogeneity of SLC6 ligands and reflect realistic overlap within activity landscapes.

Docking affinity and structural enrichment were only moderately correlated (Spearman ρ ≈ 0.5), indicating partial complementarity between these metrics. This observation justified the normalized rank-based composite scoring approach, which prioritized compounds achieving balanced performance across both dimensions. Such integration reduces bias arising from individual scoring artifacts and improves robustness of candidate selection. ZINC67840571 exemplifies this principle, combining strong predicted binding with acceptable structural enrichment.

The results demonstrate that incorporating receptor conformational diversity in docking improves the robustness and interpretability of virtual screening for GAT-1 inhibitors. Among the evaluated conformations, 7SK2 and 7Y7Z consistently achieved the best performance across global discrimination metrics (ROC AUC and KS), while other conformations showed either reduced performance or near-random behavior.

Although certain conformations (e.g., 7Y7V) exhibited higher early enrichment (EF10), this was not accompanied by strong overall discrimination, indicating that such enrichment may be driven by localized ranking effects rather than consistent separation between active and inactive compounds. This highlights the importance of combining multiple evaluation metrics when selecting receptor conformations for ensemble docking.

The two-conformation mean ensemble (7SK2 and 7Y7Z) provided the best overall performance, achieving improved ROC AUC and KS statistics compared to individual conformations. Importantly, this improvement was obtained without introducing additional model complexity or weighting schemes, supporting the use of simple averaging as a robust and interpretable aggregation strategy.

Candidate prioritization was further stabilized by combining docking ensemble scores with structural enrichment. The resulting rankings were largely consistent across different conformer aggregation strategies, with a subset of candidates exhibiting low rank variability (rank range ≤ 10), indicating robustness of prioritization.

Notably, the highest-ranked compound (ZINC03643214) corresponds to a previously reported GAT-1 inhibitor candidate, providing external validation of the proposed workflow. Importantly, the method also identified ZINC67840571 as the top novel candidate, demonstrating its ability to generalize beyond known compounds and propose structurally relevant new candidates.

Overall, the proposed framework balances predictive performance, robustness, and interpretability and is readily applicable to other flexible membrane protein targets where single-structure docking may be insufficient.

Although the re-docking of tiagabine into the 7Y7Z structure yielded higher RMSD values (2–3 Å), indicating reduced reproducibility of the crystallographic pose, this conformation was retained for candidate ranking due to its complementary statistical and structural properties. Specifically, 7Y7Z achieved strong discrimination between active and inactive compounds (ROC AUC = 0.727, KS = 0.490), comparable to the best-performing conformer 7SK2. Moreover, structural analysis revealed that 7Y7Z represents a receptor state with a larger binding-site volume and reduced steric hindrance, features that are particularly relevant to accommodating diverse candidate ligands. To mitigate the limitations of individual conformers, we employed an ensemble docking strategy that when combined yielded the highest overall discrimination (ROC AUC = 0.732, KS = 0.539). Thus, while 7Y7Z alone showed weaker redocking reproducibility, its complementary statistical and structural contributions justified its inclusion in the ensemble, ultimately improving the robustness of candidate prioritization.

ADMET analysis further refined prioritization. One cluster, characterized by higher lipophilicity, improved predicted blood–brain barrier permeability, and acceptable CNS penetration parameters, contained both principal candidates.

Several limitations should be emphasized. Docking scores provide relative ranking rather than quantitative affinity prediction. The positive reference dataset size may constrain enrichment statistics. Finally, experimental validation is required to confirm functional inhibition and subtype selectivity across SLC6 family members.

The integration of pharmacophore modeling, ISM filtering, molecular docking, structural enrichment analysis, and ADMET profiling enabled multi-criteria prioritization of GAT-1 inhibitor candidates. The workflow increases selectivity toward pharmacologically and pharmacokinetically plausible CNS-active ligands while reducing reliance on individual scoring paradigms. This strategy is readily transferable to other membrane transporters and central nervous system drug discovery targets.

In conclusion, we developed a multi-layered in silico workflow for prioritizing novel GAT-1 inhibitor candidates from the ZINC natural product collection by integrating pharmacophore-based screening, ISM-SM/EIIP filtering, molecular docking, structural enrichment, and ADMET profiling of known active compounds. The extension of docking from a single receptor structure to a multi-conformation GAT-1 ensemble, combined with calibration using experimentally derived active and negative reference sets, substantially strengthened the structure-based component of the approach, with the weighted ensemble consistently outperforming individual conformations and highlighting the importance of receptor flexibility. At the candidate level, integration of the docking ensemble score with structural enrichment through percentile rank normalization enabled robust and interpretable prioritization, without assumptions about score distributions. The resulting rankings were highly consistent across different conformer aggregation strategies, with strong rank correlations and overlap of top candidates, indicating that prioritization was not driven by a single representation choice. A stable core of top-ranked molecules was identified, combining strong docking support, favorable structural enrichment, and low sensitivity to conformer handling. Overall, this workflow provides a robust computational framework for GAT-1 hit prioritization and identifies a set of reliable candidates for future experimental validation, while offering a transferable consensus strategy for other flexible membrane protein targets where single-structure docking is insufficient to capture the full binding landscape.

The multi-tiered computational workflow presented here illustrates how rational drug design principles can be applied to challenging CNS targets such as GAT-1. By integrating pharmacophore modelling, ISM, ensemble docking, and structural enrichment, we established a systematic pathway that mirrors modern drug discovery pipelines. While experimental validation remains essential, the present study demonstrates how computational strategies can reduce attrition rates by prioritising ligands that are both binding-compatible and structurally selective. Although molecular dynamics simulations are a common strategy for accounting for receptor flexibility, in this study, we addressed conformational variability by employing ensemble docking across multiple experimentally resolved GAT-1 structures. This approach allowed us to capture diverse binding site geometries without introducing the additional complexity and computational cost of MD, thereby providing a pragmatic balance between accuracy and efficiency for large-scale virtual screening.

## 4. Materials and Methods

### 4.1. ZincPharmer

ZINCPharmer (http://zincpharmer.csb.pitt.edu), accesed on 1 March 2026 [17] is an online interface for searching purchasable compounds from the ZINC database using Pharmer pharmacophore search technology.

### 4.2. ISM-SM/EIIP Filtering

In this work, we analyze the GAT-1 protein using the Informational Spectrum Method for Small Molecules (ISM-SM) [18,19]. The protocol for this approach consists of several steps. First, a sequence (protein or DNA) is converted into a signal by assigning numerical values to each element (amino acid or nucleotide). These values correspond to the electron–ion interaction potential (EIIP), which determines the electronic properties of amino acids and nucleotides and is essential for their intermolecular interactions. The EIIP descriptors [30,31] are easily calculated using the following formulas:

(1) $$W=0.25\frac{Z^{*}\times \sin\left(1.04\pi Z^{*}\right)}{2\pi },$$

(2) $$Z^{*}=\frac{1}{N}\sum_{i=1}^{m}n_{i}Z_{i}$$

where i is the type of the chemical element, Z is the valence of the i-th chemical element, n is the number of the i-th chemical element atoms in the compound, m is the number of types of chemical elements in the compound, and N is the total number of atoms.

The EIIP signal is transformed using a discrete Fourier transform (DFT) to obtain the informational spectrum (IS) as a representation of a sequence in the form of a series of frequencies and amplitudes. In practical calculations, the DFT was evaluated using the Fast Fourier Transform (FFT) algorithm, which provides an efficient implementation of the same transformation.

(3) $$X\left(n\right)=\sum_{m=1}^{N}x\left(m\right)e^{-\frac{i2\pi n\left(m-1\right)}{N}},n=1,2,\ldots ,\frac{N}{2}$$

where m is the summation index; x(m) is the m-th element of the EIIP numerical sequence, i.e., the m-th member of a given numerical “signal” series (from a transformed, encoded primary protein sequence in our case); N is the sequence length (the total number of points in this series); n is the number of a discrete frequency (ranging from 1 on up to N/2) in the DFT; X(n) are the discrete Fourier transformation amplitude coefficients corresponding to each discrete frequency n; and 2π × (n/N) is the phase angle at each given m in the amino acid series of the protein in question.

The corresponding informational spectrum is represented by the energy density spectrum:

(4) $$S\left(n\right)=X\left(n\right)*X\left(n\right)={\left|X\left(n\right)\right|}^{2},\;n=1,2,\ldots ,N/2$$

where X*(n) denotes the complex conjugate of X(n).

In ISM, sequences are analyzed as discrete signals with unit spacing d = 1. Therefore, the maximum frequency is 0.5, and the frequency resolution is 1/N. The n-th spectral component corresponds to the normalized frequency f(n) = n/N. Because different proteins and ligands have different sequence lengths N, the corresponding DFT frequency indices are associated with different spectral resolutions. Therefore, frequencies are represented in normalized form f = n/N, yielding a common frequency interval. For real-valued signals, the spectrum is symmetric, and only the frequency range 0 ≤ f ≤ 0.5 contains unique information. Consequently, informational spectra are represented as a series of normalized frequencies 0 ≤ f ≤ 0.5 and their corresponding amplitudes.

With this, the virtual spectroscopy method is feasible for functionally analyzing protein sequences without prior experimental data. A small molecule is imported in SMILES notation and decoded by atomic groups into an array of corresponding EIIP values. Using FFT, the corresponding IS of a small molecule is computed. This spectrum is further multiplied by the protein receptor’s IS to obtain the cross-spectrum (CS). The cross-spectral function characterizes the frequency-domain properties of two signals. For a discrete series, it is defined as follows:

(5) $$C\left(j\right)=\prod_{i=1}^{N}S\left(i,j\right)$$

where S(i,j) denotes the j-th element of the i-th informational spectrum, and C(j) is the corresponding cross-spectral component.

From common frequencies in CS, one can determine whether a protein interacts with small molecules and identify the corresponding binding region, because overlapping frequencies in spectra indicate long-range interactions. These frequencies correspond to conserved sequence domains, so when a ligand exhibits the same dominant frequency as the protein, it indicates potential binding compatibility at that domain.

### 4.3. Receptor Preparation

All available GAT-1 receptor structures were taken from the RCSB database, with the following PDBID codes: 7SK2 [32], 7Y7V, 7Y7W, 7Y7Y, and 7Y7Z [33]. The corresponding sequence was taken from the UNIPROT database [34]: code P30531 sodium- and chloride-dependent GABA transporter 1, gene name SLC6A1.

### 4.4. Data Generation

Ligands for the learning sets were downloaded from the BindingDB [35] database, including the receptor ID ChEMBL3371, which contains 219 active compounds with corresponding 63 Ki, 6 Kd, and 150 reported IC50 values. The learning set was split into positive and negative sets using the criterion IC50 < 100 uM for the positive set, while the remaining compounds were used to construct the negative dataset. This boundary was chosen as, above this value, all other measurements are reported as >100 uM, i.e., without significant activity. Standard drugs targeting serotonin (P31645, SCL6A4), dopamine (Q01959, SLC6A3), and noradrenaline transporter (P23975, SCL6A2) were downloaded from the ChEMBL database [36] and added to the negative set. These compounds were added to ensure structural criteria for pure GAT-1 inhibitors and to avoid multitargeting towards other transporters. After removing all duplicates, this yielded a positive set of 65 compounds and a negative set of 136 compounds. Molecules were converted from SMILES to sdf format and protonated at physiological pH 7.4.

### 4.5. Molecular Docking

The receptor structures were protonated at physiological pH 7.4. The binding site for molecular docking was defined as a box with dimensions 18 × 18 × 18 Å, centered on the centroid of the co-crystallized ligand atoms. The coordinates (x, y, z) of grid boxes for all structures are as follows: 7SK2 (112.35, 103.81, 104.08); 7Y7W (104.35, 106.81, 107.08); 7Y7V (104.35, 110.81, 111.08); 7Y7Y (104.35, 106.81, 107.08); and 7Y7Z (101.35, 104.81, 104.08). The number of modes was set to nine, and exhaustiveness to 50. The scoring function used was Autodock Vina empirical scoring function. The docking protocol was validated by redocking of the co-crystallized ligands for all receptor structures.

Virtual screening was performed in VEGA ZZ 3.2.4.29 [37], with the aid of Autodock Vina 1.1.2 [38]. 

### 4.6. Multi-Conformation Docking and Consensus Scoring

To account for receptor flexibility and improve the robustness of docking-based prioritization, molecular docking was performed across multiple experimentally resolved conformations of the human GAT-1 transporter. In addition to the primary cryo-EM structure (PDB ID 7Y7Z), four additional conformations (PDB IDs: 7SK2, 7Y7V, 7Y7Y, and 7V7W) were included in the analysis. All structures were prepared under identical conditions, as described in Section 4.5.

The positive (active) and negative (decoy) reference sets described in Section 4.4 were docked independently into each receptor conformation. For each molecule and each conformation, a docking score (binding energy) was obtained. Since docking energies are conformation-dependent and may differ in scale across receptor structures, scores were transformed into activity-like values by sign inversion (−ΔG), such that higher values corresponded to stronger predicted binding.

To evaluate the ability of each receptor conformation to discriminate between active and decoy compounds, receptor-specific docking performance was assessed using receiver operating characteristic area under the curve (ROC AUC), the Kolmogorov–Smirnov (KS) statistic, and an enrichment factor of 10% of the ranked list (EF10%). These metrics were used to compare the discriminatory utility of the five conformations.

The ROC AUC evaluates the ability of a scoring function to discriminate between the POS and NEG compound sets. After class labels are assigned based on the experimental activity threshold (100 μM), the decision threshold applied to the docking score is varied across the full range of observed score values. For each threshold, sensitivity and specificity are calculated, generating the ROC curve. The ROC AUC is then computed as the area under this curve and provides a threshold-independent measure of discrimination performance.

Based on this evaluation, together with structural interpretation of the receptor states, conformations 7SK2 and 7Y7Z were selected for downstream ensemble docking. A simple ensemble docking score was then defined as the mean of normalized rank activity scores across these two conformations:

*Dock_ensemble_*(c) = (*NRank_7SK2_*(c) + *NRank_7Y7Z_*(c))/2 (6)

where *NRank_7SK2_*(c) and *NRank_7Y7Z_*(c) denote normalized rank activity scores of candidate c in the two selected conformations. This formulation avoids heuristic performance-based weighting and provides a stable consensus score based on the two receptor conformations showing the strongest and most consistent discrimination between the positive and negative reference compounds.

#### Conformer Aggregation Strategies and Robustness Analysis

To assess the robustness of candidate prioritization with respect to ligand conformer handling, three aggregation strategies were evaluated: (1) best: lowest docking energy across conformers; (2) top-five: mean energy of the five best conformers; (3) clean: single representative conformer per molecule.

Candidate rankings obtained from these strategies were compared using (1) Spearman rank correlation; (2) Pearson correlation; and (3) Jaccard index of top-ranked candidates.

For the latter analysis, let TopK_A and TopK_B denote the sets of the top K candidates obtained by methods A and B, respectively. The overlap count was defined as (|TopK_A ∩ TopK_B|), while the union count was defined as (|TopK_A ∪ TopK_B|). The Jaccard index was then calculated as the ratio of overlap count to union count.

Candidates were defined as stable if their rank variation across strategies did not exceed 10 positions: rank_range ≤ 10.

### 4.7. ADMET Calculations

The pharmacokinetic properties of the compounds were calculated using the pkCSM server [39] (https://biosig.lab.uq.edu.au/pkcsm/prediction, accessed on 1 March 2026), with 36 total variables. A detailed interpretation of the calculated ADMET properties is available at https://biosig.lab.uq.edu.au/pkcsm/theory (accessed on 1 March 2026). In the case of the non-numerical prediction results Yes/No, for further calculations, they were converted to 1 and 0, respectively. The resulting data were subjected to k-means clustering. The number of clusters was initially set between 2 and 5, with the clustering criterion based on the determinant (W). Initial partitions were randomized with 10 repetitions, and clustering was stopped after 500 iterations or upon convergence at 0.00001. To determine the optimal number of clusters, an elbow analysis was performed by plotting the within-cluster sum of squares against the number of clusters. The clustering was done in XLSTAT 2026.1.0 Trial version [40].

### 4.8. Structural Similarity Analysis and Candidate Ranking

#### 4.8.1. Molecular Representations

- Binary molecular fingerprints, encoding the presence or absence of predefined structural subpatterns. These included standard cheminformatics fingerprints, e.g., MACCS structural keys, PubChem fingerprints, and ECFP6 circular fingerprints [41,42], represented as binary vectors.
- Continuous molecular descriptors, consisting of numerical physicochemical and structural features, RDKit and PaDEL 1D/2D (e.g., topological indices, fragment counts, and other molecular properties) [41,43]. These descriptors were treated as continuous-valued vectors.

Binary fingerprints capture discrete structural motifs, while continuous descriptors capture quantitative molecular properties, providing complementary representations of molecular similarity. This combination is standard practice in cheminformatics similarity analysis [44].

#### 4.8.2. Distance Metrics

Molecular similarity was quantified using standard distance metrics appropriate for each feature type:

- Tanimoto (Jaccard) distance for binary fingerprints, defined as
  *d*_T_(A,B) = 1 − |A∩B|/|A∪B| (7)

This is the most widely used similarity metric for binary molecular fingerprints [45,46,47,48].

- Euclidean distance for continuous descriptors:

*d*_E_(A,B) = ‖A − B‖_2_ (8)

- Cosine distance for continuous descriptors:
  *d*_C_(A,B) = 1 − A⋅B/(‖A‖‖B‖) (9)

Euclidean distance captures absolute magnitude differences, while cosine distance measures angular similarity independent of vector magnitude. Both are widely used in molecular descriptor analysis [46,48].

#### 4.8.3. Structural Separation Analysis

To quantify structural separation between the positive (POS) and negative (NEG) reference sets, pairwise distance matrices were computed for all molecules in the reference set.

Distances were partitioned into

- Intra-class distances: distances between molecules within the same class (POS–POS and NEG–NEG);
- Inter-class distances: distances between molecules across classes (POS–NEG).

Mean intra-class distance was defined as

*d_intra_* = (mean(*d*_X∈POS,Y∈POS_(X,Y)) + mean(*d*_X∈NEG,Y∈NEG_(X,Y)))/2 (10)

Mean inter-class distance was defined as

*d_inter_* = mean(*d*_*X*∈POS, Y∈NEG_(X,Y)) (11)

Structural separation was quantified using the following metrics:

- Inter–intra distance ratio:

*d*_ratio_= *d*_inter_/*d*_intra_ (12)

Higher values indicate stronger separation.

- Normalized inter–intra difference:

*d*_norm_ = (*d*_inter_ − *d*_intra_)/(*d*_inter_ + *d*_intra_) (13)

This provides a scale-independent separation measure bounded between −1 and 1.

- Silhouette coefficient

The silhouette coefficient measures how well each molecule is separated from the opposite class relative to its own class [49]. It is defined as

*s* = (*b* − *a*)/max(*a*,*b*) (14)

where a is the mean intra-class distance, and b is the mean distance to the nearest opposite class. The silhouette coefficient ranges from −1 to 1, where higher values indicate stronger separation between classes, values near zero indicate overlapping clusters, and negative values indicate potential misclassification.

- Kolmogorov–Smirnov statistic

The Kolmogorov–Smirnov (KS) statistic measures the maximum difference between cumulative distributions of inter-class and intra-class distances [50]. Higher KS values indicate greater distributional separation.

#### 4.8.4. Candidate Enrichment Analysis

To evaluate whether candidate molecules are structurally closer to positive than to negative molecules, distances between each candidate and each reference molecule were computed.

The distance between candidate c and a reference group (POS or NEG) was defined as the mean distance:

d(c,G) = ∑_g∈G_ d(c,g)/∣G∣ (15)

for all candidates c∈CAND, where d(c,g) denotes the chosen molecular distance metric, and G is the POS or NEG reference set.

Structural enrichment toward the positive set was quantified using

- Gain difference:

gain_mean(c) = d(c,NEG) − d(c,POS)(16)

Positive values indicate that candidates are closer to POS. Accordingly, candidates with higher gain_mean values are considered structurally closer to the positive reference set relative to those with lower values.

### 4.9. Composite Ranking of Candidates and Final Consensus Prioritization

To integrate docking affinity and structural enrichment into a single prioritization metric, a rank-based composite score was constructed. Docking ensemble scores obtained from the two-conformation mean ensemble (Section 4.6) were ranked in ascending order after conversion to activity-like normalized rank scores, while structural enrichment scores (gain_mean, computed using cosine distance because it provided the strongest separation between positive and negative reference sets) were ranked in descending order.

To ensure scale independence and robustness to outliers, ranks were converted to normalized ranks in the interval [0,1], where 1 corresponds to the best-performing candidate for a given metric:

NRank_x_(c) = 1 − (rank_x_(c) − 1)/(N − 1) (17)

where N is the total number of screened candidates (N = ∣CAND∣), and rank(c) is the ranking position of candidate ccc, ranging from 1 (best candidate) to N (worst candidate). The transformation converts ranking positions into normalized rank-based scores in the interval [0,1].

The composite score was defined as a weighted average of percentile ranks:

Ensemble_nrank_(c) = w⋅NRank_dock_(c) + (1 − w)⋅NRank_gain_(c) (18)

where NRank_dock_(c) is the normalized rank of the docking ensemble score, NRank_gain_(c) is the normalized rank of the cosine-based structural enrichment score gain_mean, and w ∈ [0,1] controls the relative contribution of docking and structural enrichment components. In the present study, equal weighting (w = 0.5) was used unless otherwise specified.

This rank-based approach avoids assumptions about score distributions, is invariant to scale differences between metrics, and provides a robust, interpretable framework for multi-criteria candidate prioritization commonly used in cheminformatics and bioinformatics ranking analyses [51,52].

To assess the robustness of the final prioritization, rankings obtained from the clean, best-conformer, and top-N aggregation strategies were compared using Spearman rank correlation, Pearson correlation of final scores, and overlap of top-ranked candidate subsets. For the top-N conformer aggregation strategy, we used N = 5 (top-five), where the representative score of each molecule was defined as the mean docking energy of its five lowest-energy conformers. This choice was supported by the conformer distribution in the dataset, where 93.7% of molecules had five or fewer conformers (median = 2), ensuring that top-five captures the full conformational variability for the vast majority of compounds. A consensus ranking stability analysis was additionally performed by computing the mean rank, rank standard deviation, and rank range across the three aggregation strategies. To further operationalize ranking stability, candidates were classified as stable if their rank range across conformer aggregation strategies (clean, best, top-N) did not exceed 10 positions (rank_range ≤ 10). This threshold was chosen to identify candidates with low sensitivity to conformer representation while allowing for minor ranking fluctuations.

The overall computational workflow is summarized in Supplementary Figure S1. First, a reference set of known GAT-1 inhibitors was assembled and used to generate and validate a pharmacophore model and to perform enrichment analysis. Pharmacophore-based virtual screening of the ZINC database yielded 9905 candidate molecules, which were subsequently filtered using the Information Spectrum Method (ISM-SM) at the characteristic frequency F = 0.190 and AQVN/EIIP molecular descriptor criteria, resulting in 237 candidates. These compounds were then evaluated using ensemble molecular docking against five experimentally determined GAT-1 conformations and by structural enrichment analysis based on similarity to known active compounds. The best-performing receptor conformations were selected using ROC analysis, and docking affinity and structural enrichment scores were integrated into a composite normalised rank score to prioritise candidates. Finally, ranking robustness was assessed across alternative conformer-aggregation strategies, and the highest-priority compounds were subjected to ADMET and drug-likeness evaluations to identify final candidates for experimental validation.

## Figures and Tables

**Table 1:** RMSD values of co-crystallized ligands redocked with individual GAT-1 conformations.

Receptor	Ligand	RMSD (Å)
7Y7Z	Tiagabine	2.6809
7SK2	Tiagabine	0.7952
7Y7Y	Nipecotic acid	1.0744
7Y7V	-	-
7V7W	GABA	2.6821

**Table 2:** Discriminative performance of individual GAT-1 conformations and the two-conformation ensemble. Performance of five GAT-1 receptor conformations (7Y7Z, 7SK2, 7Y7Y, 7Y7V, 7V7W) in distinguishing between positive and negative reference compounds, as evaluated using ROC AUC, the Kolmogorov–Smirnov (KS) statistic, and an enrichment factor of 10% (EF10%). The final row shows the performance of the two-conformation mean ensemble (7SK2 + 7Y7Z).

Receptor	ROC AUC	KS	EF 10%
Conf 7Y7Z	0.727	0.490	1.144
Conf 7SK2	0.726	0.525	1.144
Conf 7Y7Y	0.683	0.402	1.717
Conf 7Y7V	0.680	0.361	2.861
Conf 7V7W	0.457	0.133	0.429
Ens mean 7SK2 7Y7Z	0.732	0.539	1.144

**Table 3:** Structural separation between positive and negative reference compounds.

Distance	Silhouette Coefficient	Inter–Intra Ratio	Inter–Intra Norm	Kolmogorov–Smirnov Statistic
fp_maccs:jaccard	0.071	1.213	0.096	0.098
fp_pubchem:jaccard	0.064	1.147	0.068	0.072
fp_ecfp:jaccard	0.064	1.136	0.064	0.069
vec:cosine	0.123	1.202	0.092	0.173
vec:euclidean	0.029	1.083	0.040	0.093

**Table 4:** Final ranking of top candidate molecules based on docking ensemble score and structural enrichment. Top-ranked candidate molecules prioritized using a composite normalized rank score combining docking ensemble scores (mean of 7SK2 and 7Y7Z conformations) and structural enrichment (gain_mean). Stability metrics across conformer aggregation strategies are also shown.

Name	Final Score	PRank Dock	PRank Gain	Dock Ensemble Weighted	Gain Mean	Stable Candidate
ZINC03643214	1.0000	1.0000	1.0000	1.0000	0.1809	TRUE
ZINC19571708	0.9936	0.9915	0.9958	0.9915	0.1538	TRUE
ZINC19571702	0.9894	0.9873	0.9915	0.9873	0.1513	TRUE
ZINC67840571	0.9873	0.9958	0.9788	0.9958	0.0076	TRUE
ZINC91563519	0.9788	0.9703	0.9873	0.9650	0.0562	TRUE
ZINC12157317	0.9566	0.9492	0.9640	0.9439	−0.0028	TRUE
ZINC12157306	0.9523	0.9576	0.9470	0.9523	−0.0094	TRUE
ZINC72391731	0.9513	0.9746	0.9280	0.9661	−0.0296	TRUE
ZINC12157321	0.9502	0.9364	0.9640	0.9290	−0.0028	TRUE
ZINC12157311	0.9460	0.9449	0.9470	0.9407	−0.0094	TRUE

**Table 5:** Correlation of candidate rankings across conformer aggregation strategies. Pairwise comparison of rankings obtained using clean, best-conformer, and top-N aggregation.

Method A	Method B	Spearman Rank Rho	Spearman Rank *p*-value	Pearson Score r	Pearson Score *p*-Value
Clean	best	0.9883	8.50 × 10^−194^	0.9908	2.11 × 10^−206^
Clean	top-five	0.9927	1.11 × 10^−217^	0.9945	4.03 × 10^−232^
Best	top-five	0.9958	3.62 × 10^−246^	0.9967	3.87 × 10^−258^

**Table 6:** Overlap of top-ranked candidates across conformer aggregation strategies. Jaccard similarity and overlap statistics for top-k candidate subsets.

Top k	Method a	Method b	Overlap Count	Union Count	Jaccard
5	clean	best	4	6	0.6667
5	clean	top-five	4	6	0.6667
5	best	top-five	5	5	1.0000
5	ALL	ALL	4	6	0.6667
10	clean	best	9	11	0.8182
10	clean	top-five	10	10	1.0000
10	best	top-five	9	11	0.8182
10	ALL	ALL	9	11	0.8182
20	clean	best	19	21	0.9048
20	clean	top-five	19	21	0.9048
20	best	top-five	19	21	0.9048
20	ALL	ALL	18	21	0.8571
50	clean	best	47	53	0.8868
50	clean	top-five	49	51	0.9608
50	best	top-five	48	52	0.9231
50	ALL	ALL	47	53	0.8868

**Table 7:** Top-ranked GAT-1 transporter inhibitors, with the list of intermolecular interactions between the ligand and the amino acid residues of the GAT-1 transporter, according to Protein–Ligand Interaction Profiler [23]. H: hydrogen bond, F: π-alkyl/hydrophobic interactions.

ZINC ID	Binding Energy (kcal/mol)	TYR60	GLY63	LEU64	GLY65	ASN66	PHE98	TYR140	PHE294	TYR296	LEU300	SER302	LEU303	LEU306	ASN327	VAL323
Tiagabine	−7.3	F	-	-	H	-	-	H	-	-	F	-	F	-	-	-
ZINC03643214	−10.0	F	H	-	H	H	F	F	F+H	F	-	H	F	F	H	-
ZINC67840571	−9.4	F		H	H	-	F	-	-	-	-	-	F	F	-	F

## Data Availability

The original contributions presented in this study are included in the article/Supplementary Materials. Further inquiries can be directed to the corresponding authors.

## Supplementary Materials

The following supporting information can be downloaded at https://www.mdpi.com/article/10.3390/ph19071011/s1: Table S1. Information on positive set with calculated AQVN/EIIP values, negative set, candidate set, docking scores, ML ranking, ADMET properties, and following k-means clustering. Table S2. Final prioritized candidate set. Table S3. Full candidate ranking with docking ensemble and structural enrichment scores. Table S4. Stable top-ranked candidates defined by rank range ≤ 10 across aggregation strategies. Table S5. Comparison of candidate rankings across conformer aggregation strategies. Figure S1. The scheme of the computational workflow.

## Abbreviations

The following abbreviations are used in this manuscript:

CNS	Central nervous system
GABA	γ-aminobutyric acid
GATs	GABA transporters
GAT-1	GABA transporter 1
ISM-SM	Informational Spectrum Method for Small Molecules
AQVN	Average quasi valence number
EIIP	Electron–ion interaction potential
FFT	Fourier transform
CS	Cross-spectrum
KS	Kolmogorov–Smirnov
ADMET	Absorption, distribution, metabolism, excretion, and toxicity

## References

[1] Schousboe A., Madsen K.K., Barker-Haliski M.L., White H.S. (2014). The GABA Synapse as a Target for Antiepileptic Drugs: A Historical Overview Focused on GABA Transporters. Neurochem. Res..

[2] Bormann J. (2000). The ‘ABC’ of GABA Receptors. Trends Pharmacol. Sci..

[3] Bryson A., Reid C., Petrou S. (2023). Fundamental Neurochemistry Review: GABAA Receptor Neurotransmission and Epilepsy: Principles, Disease Mechanisms and Pharmacotherapy. J. Neurochem..

[4] Mortensen J.S., Mikkelsen A.N.L., Wellendorph P. (2024). Ways of Modulating GABA Transporters to Treat Neurological Disease. Expert Opin. Ther. Targets.

[5] Bhatt M., Gauthier-Manuel L., Lazzarin E., Zerlotti R., Ziegler C., Bazzone A., Stockner T., Bossi E. (2023). A Comparative Review on the Well-Studied GAT1 and the Understudied BGT-1 in the Brain. Front. Physiol..

[6] Mermer F., Poliquin S., Rigsby K., Rastogi A., Shen W., Romero-Morales A., Nwosu G., McGrath P., Demerast S., Aoto J. (2021). Common Molecular Mechanisms of *SLC6A1* Variant-Mediated Neurodevelopmental Disorders in Astrocytes and Neurons. Brain.

[7] Genton P., Guerrini R., Perucca E. (2001). Tiagabine in Clinical Practice. Epilepsia.

[8] Singh K., Kumar P., Bhatia R., Mehta V., Kumar B., Akhtar M.J. (2022). Nipecotic Acid as Potential Lead Molecule for the Development of GABA Uptake Inhibitors; Structural Insights and Design Strategies. Eur. J. Med. Chem..

[9] Falch E., Meldrum B.S., Krogsgaard-Larsen P. (1987). GABA Uptake Inhibitors. Synthesis and Effects on Audiogenic Seizures of Ester Prodrugs of Nipecotic Acid, Guvacine and Cis-4-Hydroxynipecotic Acid. Drug Des. Deliv..

[10] Zafar S., Jabeen I. (2018). Structure, Function, and Modulation of γ-Aminobutyric Acid Transporter 1 (GAT1) in Neurological Disorders: A Pharmacoinformatic Prospective. Front. Chem..

[11] Fabricant D.S., Farnsworth N.R. (2001). The Value of Plants Used in Traditional Medicine for Drug Discovery. Environ. Health Perspect..

[12] Drewry D.H., Macarron R. (2010). Enhancements of Screening Collections to Address Areas of Unmet Medical Need: An Industry Perspective. Curr. Opin. Chem. Biol..

[13] Bhagat K., Singh J.V., Pagare P.P., Kumar N., Sharma A., Kaur G., Kinarivala N., Gandu S., Singh H., Sharma S. (2021). Rational Approaches for the Design of Various GABA Modulators and Their Clinical Progression. Mol. Divers..

[14] McInnes C. (2007). Virtual Screening Strategies in Drug Discovery. Curr. Opin. Chem. Biol..

[15] Irwin J.J., Sterling T., Mysinger M.M., Bolstad E.S., Coleman R.G. (2012). ZINC: A Free Tool to Discover Chemistry for Biology. J. Chem. Inf. Model..

[16] Dhar T.G.M., Borden L.A., Tyagarajan S., Smith K.E., Branchek T.A., Weinshank R.L., Gluchowski C. (1994). Design, Synthesis and Evaluation of Substituted Triarylnipecotic Acid Derivatives as GABA Uptake Inhibitors: Identification of a Ligand with Moderate Affinity and Selectivity for the Cloned Human GABA Transporter GAT-3. J. Med. Chem..

[17] Koes D.R., Camacho C.J. (2012). ZINCPharmer: Pharmacophore Search of the ZINC Database. Nucleic Acids Res..

[18] Veljkovic V., Cosic I., Dimitrijevic, Lalovic D. (1985). Is It Possible to Analyze DNA and Protein Sequences by the Methods of Digital Signal Processing?. IEEE Trans. Biomed. Eng..

[19] Senćanski M. (2025). New Approach for Targeting Small-Molecule Candidates for Intrinsically Disordered Proteins. Methods Protoc..

[20] Glisic S., Arrigo P., Alavantic D., Perovic V., Prljic J., Veljkovic N. (2008). Lipoprotein Lipase: A Bioinformatics Criterion for Assessment of Mutations as a Risk Factor for Cardiovascular Disease. Proteins.

[21] Veljkovic N., Branch D.R., Metlas R., Prljic J., Vlahovicek K., Pongor S., Veljkovic V. (2003). Design of Peptide Mimetics of HIV-1 Gp120 for Prevention and Therapy of HIV Disease. J. Pept. Res..

[22] Knutsen L.J.S., Andersen K.E., Lau J., Lundt B.F., Henry R.F., Morton H.E., Nærum L., Petersen H., Stephensen H., Suzdak P.D. (1999). Synthesis of Novel GABA Uptake Inhibitors. 3. Diaryloxime and Diarylvinyl Ether Derivatives of Nipecotic Acid and Guvacine as Anticonvulsant Agents. J. Med. Chem..

[23] Adasme M.F., Linnemann K.L., Bolz S.N., Kaiser F., Salentin S., Haupt V.J., Schroeder M. (2021). PLIP 2021: Expanding the Scope of the Protein–Ligand Interaction Profiler to DNA and RNA. Nucleic Acids Res..

[24] Xu T., Chen W., Zhou J., Dai J., Li Y., Zhao Y. (2020). NPBS Database: A Chemical Data Resource with Relational Data between Natural Products and Biological Sources. Database.

[25] Nakamura K., Shimura N., Otabe Y., Hirai-Morita A., Nakamura Y., Ono N., Ul-Amin M.A., Kanaya S. (2013). KNApSAcK-3D: A Three-Dimensional Structure Database of Plant Metabolites. Plant Cell Physiol..

[26] Johnston G.A. (2013). Advantages of an Antagonist: Bicuculline and Other GABA Antagonists. Br. J. Pharmacol..

[27] Sałat K., Kulig K. (2011). GABA Transporters as Targets for New Drugs. Future Med. Chem..

[28] Timple J.M.V., Magalhães L.G., Souza Rezende K.C., Pereira A.C., Cunha W.R., Andrade E Silva M.L., Mortensen O.V., Fontana A.C.K. (2013). The Lignan (−)-Hinokinin Displays Modulatory Effects on Human Monoamine and GABA Transporter Activities. J. Nat. Prod..

[29] Forster Y.M., Green J.L., Khatiwada A., Liberato J.L., Narayana Reddy P.A., Salvino J.M., Bienz S., Bigler L., Dos Santos W.F., Karklin Fontana A.C. (2020). Elucidation of the Structure and Synthesis of Neuroprotective Low Molecular Mass Components of the *Parawixia bistriata* Spider Venom. ACS Chem. Neurosci..

[30] Veljković V. (1973). The Dependence of the Fermi Energy on the Atomic Number. Phys. Lett. A.

[31] Veljković V., Slavić I. (1972). Simple General-Model Pseudopotential. Phys. Rev. Lett..

[32] Motiwala Z., Aduri N.G., Shaye H., Han G.W., Lam J.H., Katritch V., Cherezov V., Gati C. (2022). Structural Basis of GABA Reuptake Inhibition. Nature.

[33] Zhu A., Huang J., Kong F., Tan J., Lei J., Yuan Y., Yan C. (2023). Molecular Basis for Substrate Recognition and Transport of Human GABA Transporter GAT1. Nat. Struct. Mol. Biol..

[34] Bateman A., Martin M.-J., Orchard S., Magrane M., Adesina A., Ahmad S., Bowler-Barnett E.H., Bye-A-Jee H., Carpentier D., The UniProt Consortium (2025). UniProt: The Universal Protein Knowledgebase in 2025. Nucleic Acids Res..

[35] Gilson M.K., Liu T., Baitaluk M., Nicola G., Hwang L., Chong J. (2016). BindingDB in 2015: A Public Database for Medicinal Chemistry, Computational Chemistry and Systems Pharmacology. Nucleic Acids Res..

[36] Mendez D., Gaulton A., Bento A.P., Chambers J., De Veij M., Félix E., Magariños M.P., Mosquera J.F., Mutowo P., Nowotka M. (2019). ChEMBL: Towards Direct Deposition of Bioassay Data. Nucleic Acids Res..

[37] Pedretti A., Mazzolari A., Gervasoni S., Fumagalli L., Vistoli G. (2021). The VEGA Suite of Programs: An Versatile Platform for Cheminformatics and Drug Design Projects. Bioinformatics.

[38] Trott O., Olson A.J. (2010). AutoDock Vina: Improving the Speed and Accuracy of Docking with a New Scoring Function, Efficient Optimization, and Multithreading. J. Comput. Chem..

[39] Pires D.E.V., Blundell T.L., Ascher D.B. (2015). pkCSM: Predicting Small-Molecule Pharmacokinetic and Toxicity Properties Using Graph-Based Signatures. J. Med. Chem..

[40] Addinsoft XLSTAT Statistical and Data Analysis Solution.

[41] Yap C.W. (2011). PaDEL-descriptor: An Open Source Software to Calculate Molecular Descriptors and Fingerprints. J. Comput. Chem..

[42] Rogers D., Hahn M. (2010). Extended-Connectivity Fingerprints. J. Chem. Inf. Model..

[43] Landrum G., Tosco P., Kelley B., Rodriguez R., Cosgrove D., Vianello R., Sriniker, Gedeck P., Jones G., Kawashima E. Rdkit/Rdkit: 2025_09_6 (Q3 2025) Release 2026. https://zenodo.org/records/17232453.

[44] O’Boyle N.M., Sayle R.A. (2016). Comparing Structural Fingerprints Using a Literature-Based Similarity Benchmark. J. Cheminform..

[45] Muegge I., Mukherjee P. (2016). An Overview of Molecular Fingerprint Similarity Search in Virtual Screening. Expert Opin. Drug Discov..

[46] Todeschini R., Consonni V. (2009). Molecular Descriptors for Chemoinformatics.

[47] Bajusz D., Rácz A., Héberger K. (2015). Why Is Tanimoto Index an Appropriate Choice for Fingerprint-Based Similarity Calculations?. J. Cheminform..

[48] Willett P., Barnard J.M., Downs G.M. (1998). Chemical Similarity Searching. J. Chem. Inf. Comput. Sci..

[49] Rousseeuw P.J. (1987). Silhouettes: A Graphical Aid to the Interpretation and Validation of Cluster Analysis. J. Comput. Appl. Math..

[50] Massey F.J. (1951). The Kolmogorov-Smirnov Test for Goodness of Fit. J. Am. Stat. Assoc..

[51] Li X., Wang X., Xiao G. (2019). A Comparative Study of Rank Aggregation Methods for Partial and Top Ranked Lists in Genomic Applications. Brief. Bioinform..

[52] Blanes-Mira C., Fernández-Aguado P., De Andrés-López J., Fernández-Carvajal A., Ferrer-Montiel A., Fernández-Ballester G. (2022). Comprehensive Survey of Consensus Docking for High-Throughput Virtual Screening. Molecules.

