# Supplementary figures and images for: In Silico Selection of GAT-1 Inhibitors
Source: Pharmaceuticals (Basel). 2026 Jun 29;19(7):1011. doi: 10.3390/ph19071011 (PMC13416032; doi:10.3390/ph19071011)

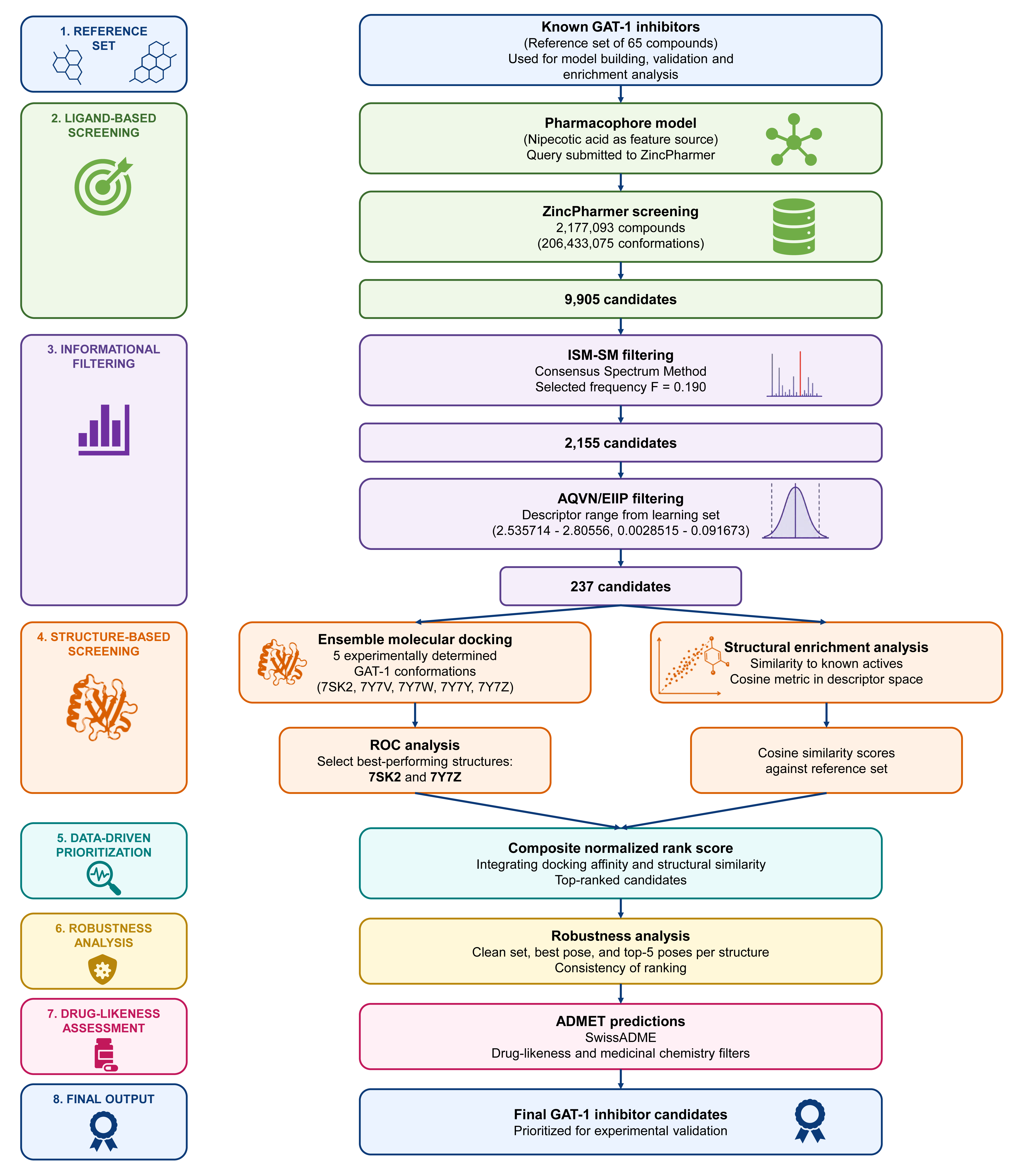

Supplement: Supplementary file 1 [file pharmaceuticals-19-01011-s001.zip › pharmaceuticals-4316397-FigureS1.png]
